# Revisiting the multidimensional interaction model of stress, anxiety and coping during the COVID-19 pandemic: a longitudinal study

**DOI:** 10.1186/s40359-022-00950-1

**Published:** 2022-11-07

**Authors:** Aleksandra M. Rogowska, Dominika Ochnik, Cezary Kuśnierz

**Affiliations:** 1 Institute of Psychology, Faculty of Social Sciences, University of Opole, pl. Staszica 1, 45-052 Opole, Poland; 2 Faculty of Medicine, University of Technology, ul. Rolna 43, 40-555 Katowice, Poland; 3 Faculty of Physical Education and Physiotherapy, Opole University of Technology, ul. Prószkowska 76, 45-758 Opole, Poland

**Keywords:** Anxiety, Coping inventory for stressful situations (CISS), Coping styles, Coping strategies, Perceived stress, The multidimensional interaction model of stress, anxiety and coping, Health psychology, Poland

## Abstract

**Background:**

Although the Multidimensional Interaction Model of Stress, Anxiety and Coping (MIMSAC) has been known for years, there is a lack of research examining this theory longitudinally in stressful events. This study aims to revisit the MIMSAC among university students during the COVID-19 pandemic.

**Methods:**

A prospective cohort study with the longitudinal design was performed during the first (W1, March 30–April 29, 2020) and second wave (W2, November 3–December 3, 2020) of the COVID-19 pandemic. A total of 216 university students with a mean age of 22 years (ranging from 20 to 36, *M* = 22.13, *SD* = 2.04) participated in the study. An online survey included Perceived Stress Scale, Coping Inventory for Stressful Situations, and Generalized Anxiety Disorder.

**Results:**

Due to the MIMSAC, all variables changed substantially across W1 and W2, adapting to an unpredictable environment. Women scored higher than men in stress, anxiety, emotion- and avoidance-oriented coping styles. We found the indirect effect of emotion-oriented coping on the stress-anxiety relationship and task-oriented coping on the anxiety-stress interaction. Avoidance was not found as a mediator in the stress-anxiety interaction.

**Conclusion:**

Emotion-oriented coping adversely affected mental health, increasing anxiety in response to stress during the COVID-19 pandemic. Task-oriented coping efficiently decreased stress in reaction to high anxiety, but only in men. Avoidance seems to be an ineffective coping style during the COVID-19 pandemic. Campus intervention programs should focus on reducing negative emotions and increasing the frequency of task-oriented coping strategies among university students.

## Introduction

**Fig. 1:**
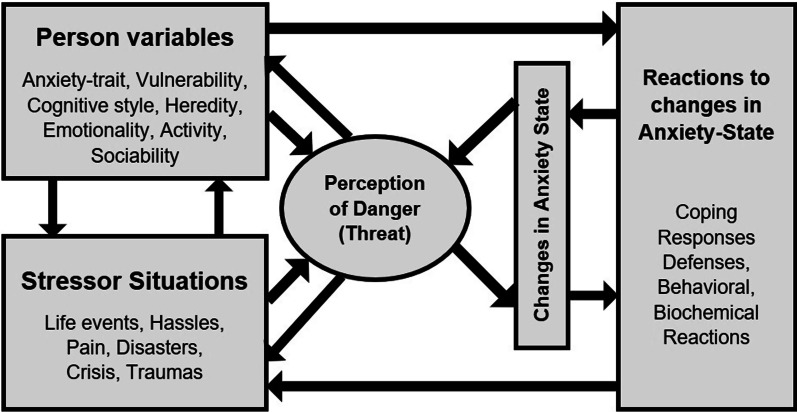
Multidimensional Interaction Model of Stress, Anxiety and Coping. *Note.* Adapted from “Stress, anxiety and coping: The multidimensional interaction model.” By N. S. Endler, 1997, *Canadian Psychology, 38*(3), 136–153. 10.1037/0708-5591.38.3.136. Copyright 1997 by the Canadian Psychological Association (CPA). Reprinted with permission

Adaptation to stressful events is based on coping strategies, facilitating or impeding mental and physical health. According to the process-oriented, Multidimensional Interaction Model of Stress, Anxiety and Coping (MIMSAC) [1, 2], an individual interacts with a given stressful situation in order to induce a perception of threat, which in turn can lead to an increase or decrease in the level of anxiety (Fig. 1). Specific behavior is one of the possible responses to a perceived dangerous situation and the resulting changes in anxiety [3, 4]. In turn, a specific coping response can affect both the variables related to the person and the situation and the perception of the situation as threatening. There are individual differences in preferred strategies and frequency of using specific styles of coping behavior. Individuals actively and consciously select and engage in particular behaviors across a wide range of various coping strategies. Endler and Parker [3] distinguished three basic coping styles: task-oriented coping (TOC, dealing with the problem by resolving it), emotion-oriented coping (EOC; focusing on negative emotions such as anger, nervousness, or sadness), and avoidance-oriented (AOC; avoiding the problem by distracting and social diversion).

Research indicated that TOC is most efficient in a controllable situation, while EOC is most effective if the situation is perceived as unalterable [2]. AOC was an appropriate reaction to stress in the short term, while TOC seems most efficient in the long term. Previous studies showed that anxiety and stress positively relate to each other [2, 4, 5]. Anxiety and stress are usually related positively to AOC and EOC styles while inversely linked with TOC [2, 4, 6–12]. There are gender differences in anxiety, perceived stress, and coping styles. Women usually report higher stress and anxiety levels and more frequently use AOC and EOC than men, while men are more likely than women to use TOC [2, 4, 13].

Although the MIMSAC was developed many years ago, little research has examined this theory longitudinally in the context of stressful events. Studies on competitive emotions among athletes showed that patterns of anxiety changed substantially at different times pre-, mid-, or post-competition due to temporal changes in stress level and selected coping strategies [14]. The present research will examine the MIMSAC [4, 5] during the first and second waves of the COVID-19 pandemic among university students.

Current research suggests that this stressful global event has an adverse consequence on the mental health of populations around the world, decreasing well-being and increasing stress, anxiety, and depression symptoms [15–21]. Benke et al. [22] indicated that greater perceived changes in life during quarantine and higher levels of restrictions regarding social contacts caused higher mental health impairments. Research has found that young adults and university or college students are at higher risk of mental disorders than other populations [9, 10, 13, 23–31]. During the COVID-19 pandemic, young adults reported experiencing lower levels of living space, occupational activity, and social contact levels, a higher extension of employment insecurity and housing conditions, and financial instability, compared to older adults [17, 32–34]. Academic stress is an additional source of stress among university students [35, 36]. Women reported a more severe overall psychological impact of the Coronavirus pandemic, including a higher level of perceived stress, anxiety, depression, and posttraumatic stress symptoms, when compared to men [9, 10, 13, 18, 23, 30, 31].

**Fig. 2:**
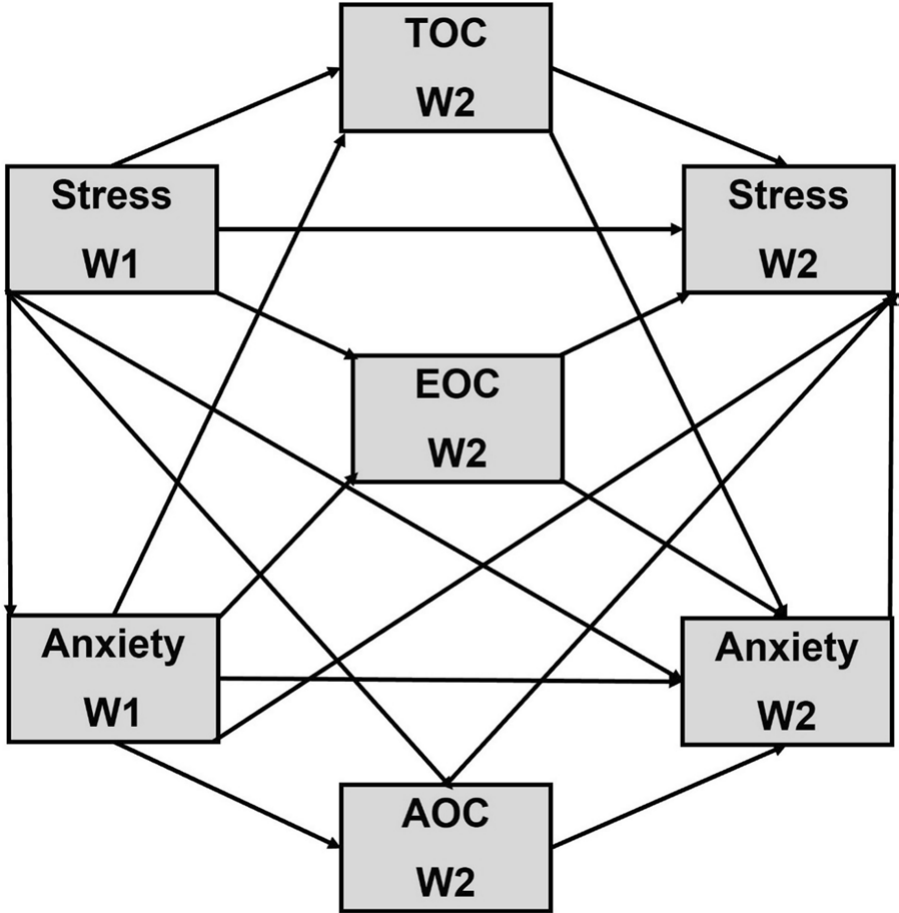
Hypothetical model of associations between stress, coping styles, and anxiety.* TOC* task-oriented coping;* EOC* emotion-oriented coping;* AOC* avoidance-oriented coping;* W1* wave 1 of the COVID-19 pandemic;* W2*wave 2 of the COVID-19 pandemic

The present study examines the MIMSAC [2] during the COVID-19 pandemic in two waves: Spring and Autumn 2020 (W1 and W2, respectively). Although some studies tested cross-sectionally the association between stress and coping styles or between anxiety and coping style, little is known about the complex model with stress, coping styles, and anxiety in a longitudinal approach. In particular, for the first time, the MIMSAC model will be tested during the pandemic. The present study will examine the structural equation model (SEM) with mediating role of coping styles (i.e., TOC, EOC, and AOC) in the relationship between stress and anxiety during the first and second waves of the COVID-19 pandemic (Fig. 2).

Schwartzer and Schultz [37] argue that acute extreme stress and cumulative exposure to aggravating daily stress over a long time may adversely affect physical and mental health. Previous research showed that university students experienced high levels of anxiety and perceived stress and frequently used emotion-oriented coping styles during the first wave of the COVID-19 pandemic [9, 13, 26, 38]. As one of the most vulnerable groups for mental disorders, university students will participate in the present study. We formulated the following research hypotheses:

1. There are significant differences in anxiety, stress, and coping styles between W1 and W2 of the COVID-19 pandemic due to dynamic changes in the environment related to restriction levels and the spread of infection.
2. Women scored higher than men in stress, anxiety, emotion- and avoidance-oriented coping styles, while they scored lower in task-oriented coping.
3. According to MIMSAC [2], coping styles play a mediating role in the relationship between stress (predictor) and anxiety (explained variable), as well as in the reciprocal association between anxiety (independent variable) and stress (dependent variable).
4. There are gender differences in the relationship between stress, coping styles, and anxiety.

## Methods

### Study design and procedure

A longitudinal study was performed during the first wave of the COVID-19 pandemic (W1) in Spring 2020 and during the second wave (W2) in Autumn 2020 at the Opole University of Technology (OUT). The Google Form was used to create the online survey with all mandatory questions to avoid missing data. University students were recruited twice through the online e-learning platform at the OUT. The invitation to participate in the study (with a link to the online survey) was presented on the e-learning Moodle platform from March 30 to April 29 during the first wave (W1) of the COVID-19 pandemic year 2020 and during the second wave (W2) between November 3 to December 3, 2020. The number of new cases of coronavirus infection in Poland ranged from 193 to 545 (*M* = 347.68) during W1, and from 5736 to 32,733 (*M* = 20,423.55) during W2. The new death cases ranged from 2 to 40 (*M* = 19.42) during W1, while from 121 to 674 (*M* = 417.84) during W2. The stringency index ranged from 57.41 to 97.04 (*M* = 83.27), and from 71.30 to 75.00 (*M* = 73.52) during W2. The Stringency Index is a composite measure of nine indicators (like closures of public transport, workplaces, and schools; stay-at-home requirements; cancellation or restrictions on public events and individual gatherings; restrictions on internal movements and international travels) calculated by the Oxford Coronavirus Government Response Tracker (OxCGRT) project [39]. The total score (ranging from 0 to 100) is calculated for each country worldwide separately as a mean of nine metrics, with higher scores indicating a stronger restriction level in the country.

Because during the data collection, remote online learning was conducted, all OUT students could participate in the study. The information about the study and informed consent was presented on the first website of the online questionnaire. Students were informed that participation is anonymous and voluntary, and they can refuse the survey at any time they want. Neither form of compensation was offered as an incentive to participate. The average time of data collection was 20 min.

The Institutional Research Board approved the study protocol (1/2020). The study followed the ethical requirements of anonymity and voluntariness of participation. Following the Helsinki Declaration, written informed consent was obtained from each student before inclusion. This study is part of an international research project, “Well-being of undergraduates during the COVID-19 pandemic: International study”, registered on the Center for Open Science (OSF) at https://osf.io/q5f4e.

### Participants

Among university students, 986 people responded to the invitation at W1 and 1354 at W2. We used the following matched criteria to compare the participants between W1 and W2: birthday and year, gender, place of residence, faculty, level, and year of the study. Those participants, whose demographic characteristics differed between W1 and W2 in at least one of these criteria, were excluded from the study. Among university students, we matched 216 individuals who participated in both W1 and W2 studies. The final sample of 216 people showed a power of 99 for ANOVA [λ = 18.00, critical *F*(1, 214) = 3.89], 99 for correlation analysis [critical *r CI* = (− 0.13, 0.13)], and 100 for linear multiple regression analysis [λ = 32.40, *F*(2, 213) = 3.04], by using G*Power software [40].

Among 216 participants, 125 were men (58%), and 91 were women (42%). The average age of the sample was 22 years (ranging from 20 to 36, *M* = 22.13, *SD* = 2.04). The place of residence reported by OUT students was a village (*n* = 104, 48%), town (*n* = 83, 38%), and city (*n* = 29, 13%). Most of the participants studied full-time (*n* = 188, 87%). The firstyear of the study represented 97 participants (45%), second-year 58 people (27%), third-year students were 43 (20%), and two of fifth-year individuals (1%). The first level of study (Bachelor’s degree) reported 162 students (75%), the second level (Master’s degree) 31 (14%), and five-years’ master’s study 23 (11%). In the sample, most persons studied at Faculty of Physical Education and Physiotherapy (*n* = 68, 32%), Electrical Engineering, Automatics and Computer Science (*n* = 59, 27%), Production Engineering and Logistics (*n* = 44, 20%), Mechanical Faculty (*n* = 66, 17%), Economics and Management (*n* = 7, 3%), and Construction and Architecture (*n* = 2, 1%).

### Measures

*The Perceived Stress Scale (PSS)* was developed by Cohen et al. [41] to assess psychological stress. PSS-10 is a self-report ten-item questionnaire with a 5-point Likert scale (ranging from 0 = *Never*, to 4 = *Very often*). Participant indicates how often he/she experienced a given type of behavior in the last month. Total scores range between 0 and 40, with higher scores indicating higher levels of perceived stress. The internal consistency of the PSS-10 is Cronbach’s α = 0.88 during W1 and α = 0.90 at W2.

*Coping Inventory for Stressful Situations (CISS)* was developed by Endler and Parker [3] and consisted of 48-items, included in three scales (16 items in each dimension): task-oriented, emotion-oriented, and avoidance-oriented coping styles. Respondents rated on a 5-point Likert scale (1 = *Not at all*, to 5 = *Very much*) the degree of engagement in various types of activity during a difficult, stressful, or upsetting situation. Higher scores are interpreted as greater use of the coping style. In the present study, reliability (Cronbach’s α ) for task- (TOC), emotion- (EOC), and avoidance- oriented coping (AOC) was 0.90, 0.90, 0.79 during W1, and 0.93, 0.91, 0.83 during W2, respectively.

*The generalized anxiety disorder (GAD-7)* scale was used to assess anxiety risk [42]. The GAD-7 is a self-reported 7-items scale to screen for anxiety symptoms following a persistent and excessive worry about various issues, according to Diagnostic and Statistical Manual of Mental Disorders, fifth edition (DSM-V) criteria. Participant rates on a 4-point Likert scale (0 = *Not at all*, 1 = *Several days*, 2 = *More than half the days*, and 3 = *Nearly every day*), how often he/she experienced anxiety symptoms during the last two weeks. Higher scores indicate higher anxiety disorder risk. The Cronbach’s α for the GAD-7 in this study was 0.91 during W1 and at 0.93 W2.

### Statistical analysis

Parametric properties of the data were examined using descriptive statistics, like mean (*M*), standard deviation (*SD*), median (*Mdn*), skewness, and kurtosis. A repeated measure two-way mixed factor analysis of variance (ANOVA) was conducted separately for each outcome measure (perceived stress, anxiety, task-, emotion- and avoidance-oriented coping style) with a 2 (Gender: Women, Men) × 2 (COVID-19 pandemic: Wave 1, Wave 2) design, to verify the hypotheses 1 and 2. The effect size was assessed by a partial eta-square (η^2^_*p*_). Associations between variables were examined using Pearson’s correlation. Finally, a structural equation model (SEM) was performed, with maximum likelihood (ML) estimation method, and bias-corrected percentile method (BCa) booptrapping technique for 2000 resamples. A bootstrap confidence interval (95% *CI*_*B*_) not exceeding “0” indicates a significant effect. The SEM model was assessed using such fit indices as χ^2^/*df* (χ^2^/*df* < 3 indicates acceptable fit), root mean square error of approximation (RMSEA < 0.08 shows a good fit), standardized root mean square residual (SRMR < 0.06 is satisfactory), and the comparative fit index (CFI > 0.95 is acceptable) [43]. Finally, to compare the SEM model between men and women, a multigroup analysis was conducted, using the plugin for AMOS [44]. Chen [45] suggests a change of ≥ − 0.005 in CFI, supplemented by a change of ≥ 0.010 in RMSEA, as an indicator of non-invariance, in the case when the compared sample sizes are unequal.

Statistical analyses were performed using JASP ver. 0.16.0.0 software [46], while SEM model and multigroup comparison were tested using AMOS ver. 26 for Statistical Package for the Social Sciences [47].

## Results

### Differences between gender and two waves of the COVID-19 pandemic

**Fig. 3:**
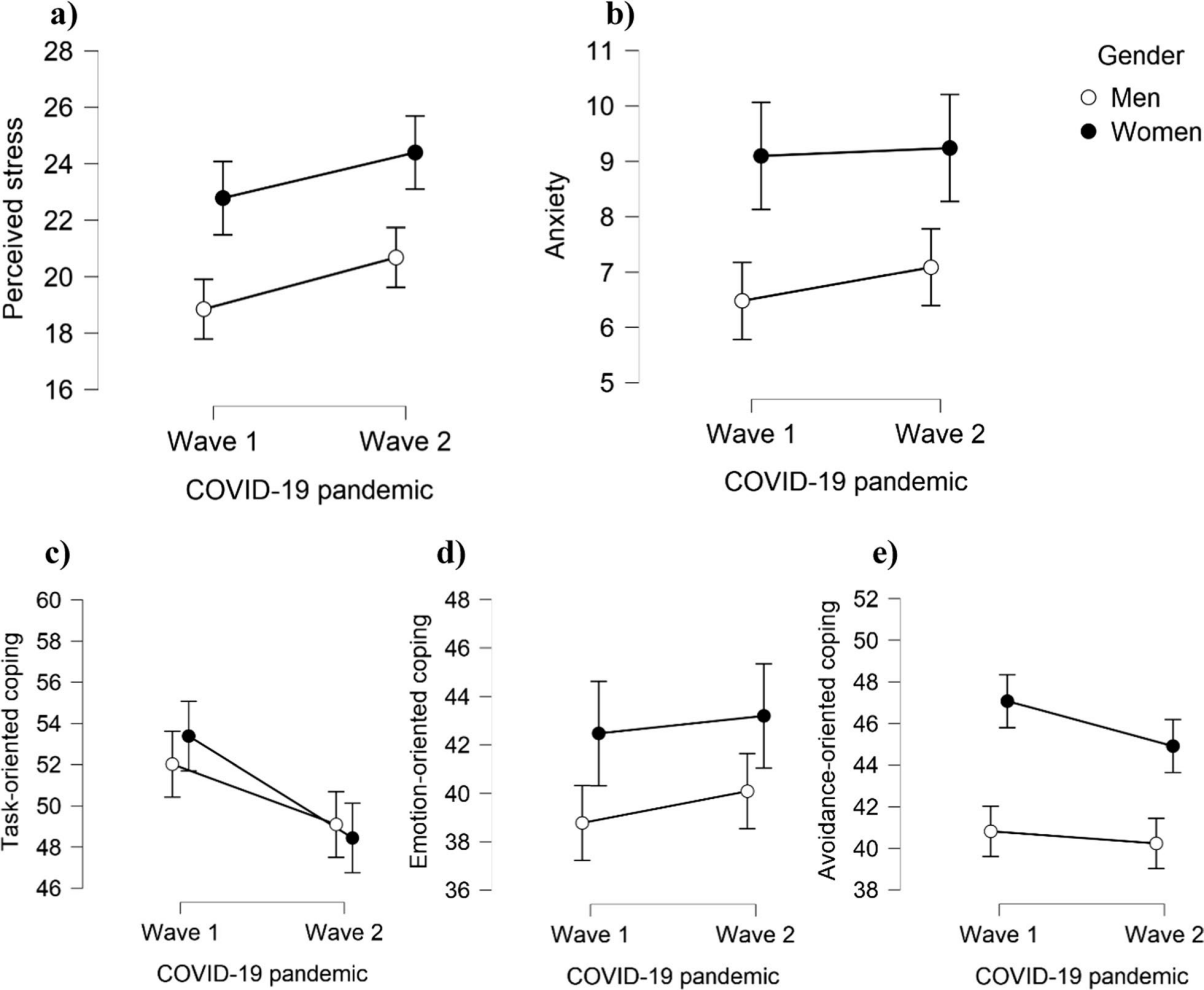
Gender differences in mean scores of** a** stress;** b** anxiety;** c** task-oriented coping;** d** emotion-oriented coping, and **e** avoidance-oriented coping styles during the first and second waves of the COVID-19 pandemic. Errors bars represent 95% confidence interval (*CI*)

The preliminary analysis was performed to examine parametric properties of perceived stress, anxiety, and coping styles. Descriptive statistics (Table 1) demonstrated good properties. Therefore, parametric tests were applied in the following steps. Both hypotheses H1 and H2 were examined using a repeated measure two-way mixed factor ANOVA. The simultaneous effects of gender and pandemic wave (as factor variables) on stress, anxiety, and coping strategies were tested separately for each dependent variable (Table 2; Fig. 3).

**Table 1:** Descriptive statistics

	Total sample (*n* = 216)							
	Min.	Max.	Range	*M*	*Mdn*	*SD*	Skewness	Kurtosis
Perceived stress W1	0	39	39	20.505	22	8.614	–0.203	–0.671
Anxiety W1	0	21	21	7.583	7	5.349	0.572	–0.295
Task W1	31	79	48	52.597	54	10.488	–0.044	–0.352
Emotion W1	16	73	57	40.333	40	12.45	0.227	–0.695
Avoidance W1	18	65	47	43.454	44	9.643	–0.147	–0.512
Perceived stress W2	2	38	36	22.245	23	7.86	–0.245	–0.688
Anxiety W2	0	21	21	7.995	7	5.789	0.455	–0.85
Task W2	17	78	61	48.819	48.5	11.918	0.024	–0.242
Emotion W2	16	71	55	41.398	42	12.911	–0.048	–0.907
Avoidance W2	16	66	50	42.204	43	10.405	–0.174	–0.541
*Women (n = 91)*								
Perceived stress W1	3	39	36	22.78	24	8.043	–0.624	0.23
Anxiety W1	0	21	21	9.099	9	5.123	0.588	–0.199
Task W1	32	76	44	53.385	55	10.06	0.06	–0.259
Emotion W1	16	69	53	42.473	42	12.595	0.026	–0.783
Avoidance W1	26	64	38	47.077	48	8.078	–0.186	–0.144
Perceived stress W2	6	38	32	24.396	25	7.151	–0.423	–0.236
Anxiety W2	0	21	21	9.242	8	5.604	0.396	–0.976
Task W2	17	78	61	48.44	48	10.854	0.069	0.302
Emotion W2	18	71	53	43.198	44	13.651	–0.147	–0.897
Avoidance W2	21	66	45	44.912	46	9.234	–0.448	0.191
*Men (n = 125)*								
Perceived stress W1	0	37	37	18.848	19	8.668	0.089	–0.789
Anxiety W1	0	21	21	6.48	6	5.259	0.685	–0.249
Task W1	31	79	48	52.024	54	10.793	–0.084	–0.426
Emotion W1	17	73	56	38.776	38	12.159	0.376	–0.483
Avoidance W1	18	65	47	40.816	41	9.86	0.085	–0.605
Perceived stress W2	2	36	34	20.68	21	8.01	–0.072	–0.833
Anxiety W2	0	21	21	7.088	6	5.774	0.566	–0.723
Task W2	22	78	56	49.096	50	12.672	–0.014	–0.504
Emotion W2	16	67	51	40.088	41	12.234	–0.031	–0.944
Avoidance W2	16	66	50	40.232	40	10.795	0.075	–0.668

Results showed statistically significant differences between the first and second waves of the COVID-19 pandemic in task-oriented coping (W1 > W2, with medium effect size), perceived stress (W1 < W2, with small effect size), and avoidance-oriented coping styles (W1 > W2, with small effect size). No differences were found between W1 and W2 in anxiety and emotion-oriented coping style. Gender showed a medium effect on stress, anxiety, and avoidance coping and a small effect on emotion-oriented coping. As it is presented in Table 2; Fig. 3, women scored significantly higher than men in all these variables. Although some gender differences were found in task-oriented coping at W1 and W2, it was statistically insignificant. Interaction effect between gender and pandemic was not found (*p* > 0.05).

**Table 2:** Two-way ANOVA statistics for study variables

	Women (*n* = 91)		Men (*n* = 123)		Effect	*F* (1, 214)	*p*	η _p_^2^
Variable	*M*	95% CI	*M*	95% CI				
Stress					G	16.66	< 0.001	0.07
W1	22.78	[21.04, 24.52]	18.85	[17.37, 20.33]	W	8.47	< 0.001	0.04
W2	24.40	[22.81, 25.98]	20.68	[19.33, 22.03]	G × W	0.03	0.855	0.00
Anxiety					G	14.41	< 0.001	0.06
W1	9.10	[8.02, 10.17]	6.48	[5.56, 7.40]	W	0.83	0.365	0.00
W2	9.24	[8.06, 10.42]	7.09	[6.08, 8.09]	G × W	0.32	0.574	0.00
TOC					G	0.07	0.787	0.00
W1	53.38	[51.22, 55.55]	52.02	[50.17, 53,87]	W	21.86	< 0.001	0.09
W2	48.44	[45.97, 50.91]	49.10	[46.99, 51.20]	G × W	1.43	0.232	0.01
EOC					G	5.35	0.022	0.02
W1	42.47	[39.92, 45.02]	38.78	[36.60, 40.95]	W	1.22	0.270	0.01
W2	43.20	[40.54, 45.85]	40.09	[37.82, 42.35]	G × W	0.10	0.751	0.00
AOC					G	21.85	< 0.001	0.09
W1	47.08	[45.16, 48.97]	40.82	[39.20, 42.43]	W	4.64	0.032	0.02
W2	44.91	[42.81, 47.01]	40.23	[38.44, 42.02]	G × W	1.53	0.217	0.01

* G* gender,* W* pandemic wave, G × W = interaction between gender and pandemic wave,* W1* wave 1 of the COVID-19 pandemic,* W2*  wave 2 of the COVID-19 pandemic,* TOC* task-oriented coping,* EOC* emotion-oriented coping,* AOC* avoidance-oriented coping. *N* = 216

### Association between anxiety, stress, and coping styles

**Fig. 4:**
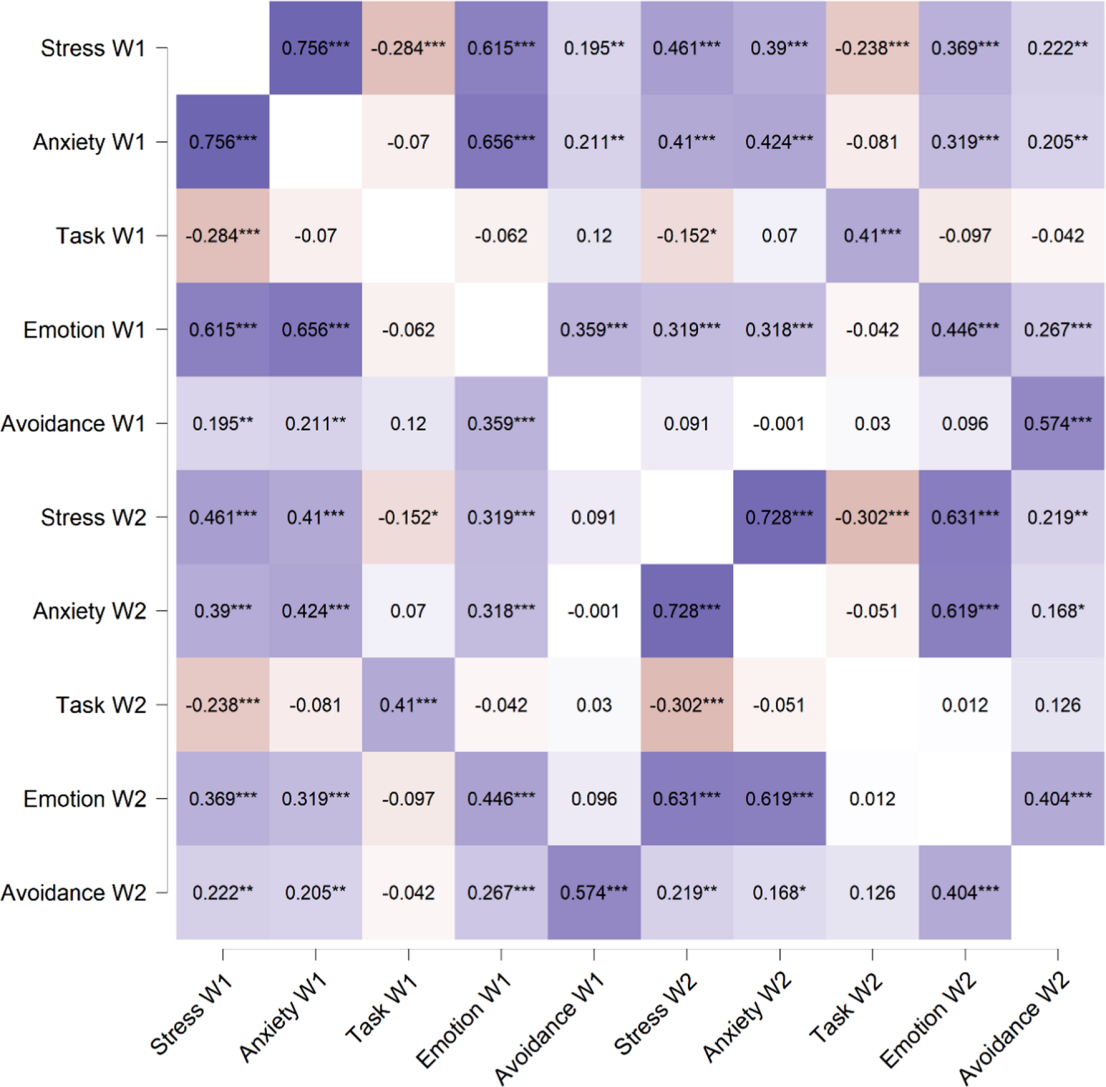
Pearson’s correlations (*r*) coefficients between perceived stress, anxiety, and coping styles (task-, emotion-, and avoidance-oriented).* W1* wave 1 of the COVID-19 pandemic,* W2* wave 2 of the COVID-19 pandemic. Violet indicated positive correlations while red indicated negative correlations. *p < 0.05, **p < 0.01, ***p < 0.001

The Pearson’s correlation was performed preliminarily to examine associations between all variables (see Fig. 4, for more details). As expected, stress W1 was positively related to anxiety W1 (*r* = 0.756, *p* < 0.001), emotion-oriented W1 (*r* = 0.615, *p* < 0.001) and avoidance-oriented coping W1 (*r* = 0.195, *p* < 0.05), and negatively to task-oriented coping W1 (*r* = − 0.284, *p* < 0.001). A positive relationships was also found between anxiety W1 and both emotion-oriented W1 (*r* = 0.656, *p* < 0.001) and avoidance-oriented coping W1 (*r* = 0.211, *p* < 0.05). No correlation was shown between anxiety W1 and task-oriented coping W1 (*r* = -0.070, *p* > 0.05). Stress W2 positively correlated with anxiety W2 (*r* = 0.728, *p* < 0.001) emotion-oriented W1 (*r* = 0.631, *p* < 0.001) and avoidance-oriented coping W1 (*r* = 0.219, *p* < 0.01), and negatively to task-oriented coping W1 (*r* = − 0.302, *p* < 0.001). Similar to W1, during W2 anxiety was positively associated with emotion-oriented W2 (*r* = 0.619, *p* < 0.001), while no relationships was found with both task-oriented W2 (*r* = − 0.051, *p* > 0.05) and avoidance-oriented coping W2 (*r* = 0.168, *p* > 0.05).

**Fig. 5:**
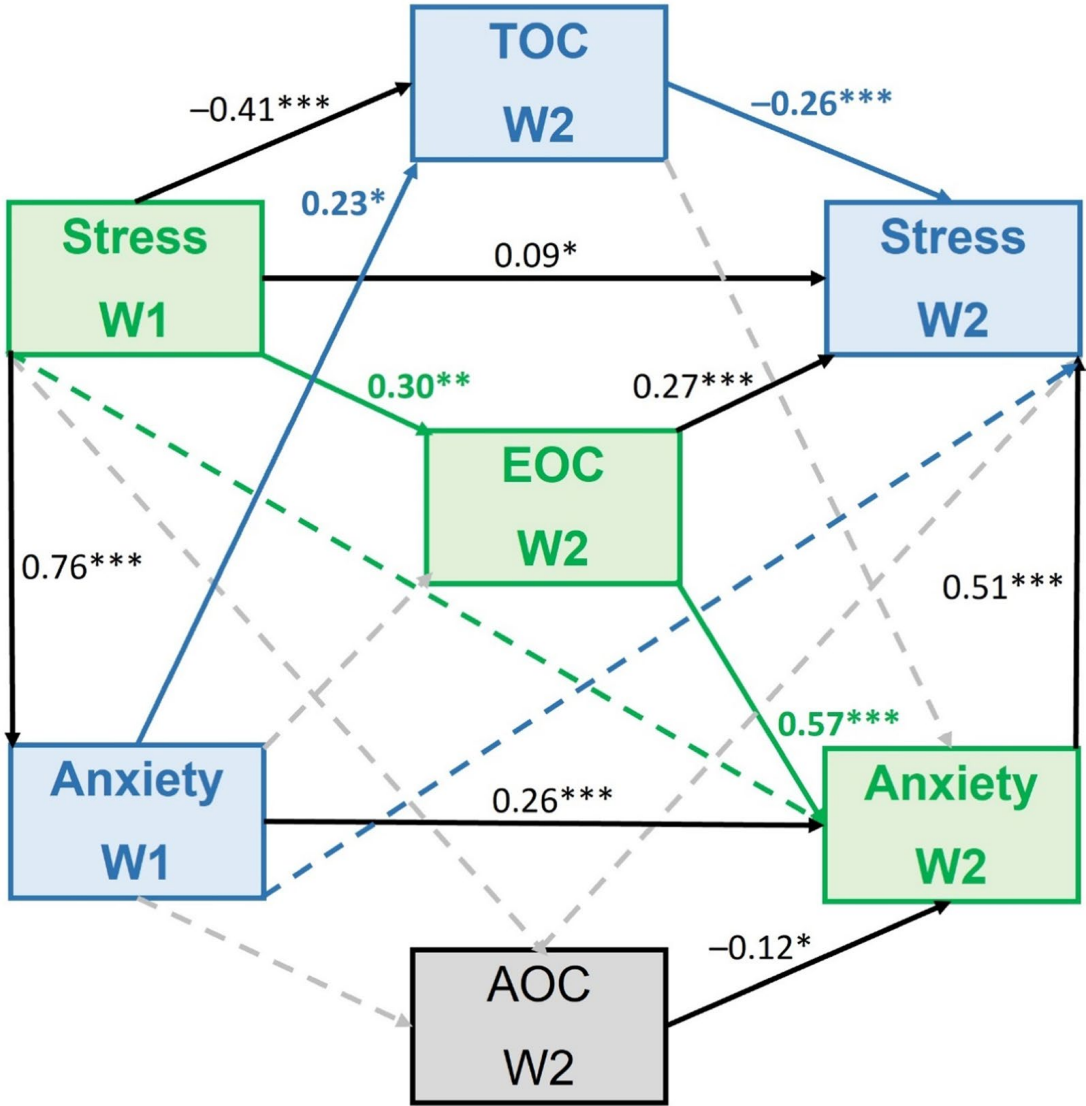
Associations between stress, coping styles, and anxiety (Model 1).* TOC* task-oriented coping,* EOC*  emotion-oriented coping,  *AOC*  avoidance-oriented coping,* W1*  wave 1 of the COVID-19 pandemic, * W2* wave 2 of the COVID-19 pandemic. Numbers represent standardized regression coefficient (β). The dashed line represents an insignificant association. The mediating effect of TOC W2 on the relationship between Anxiety W1 and Stress W2 is marked by blue color, while green color highlights the indirect effect of Stress W1 on Anxiety W2 via EOC W2. *p < 0.05, **p < 0.01, ***p < 0.001

The longitudinal analysis of the association between Stress W1 and Anxiety W1 as predictors, TOC W2 (M_1_), EOC W2 (M_2_), and AOC W2 (M_3_) as mediators, and also Stress W2 and anxiety W2 as explained variables, were examined using SEM Model 1 (Fig. 2). Results are presented in Table 3; Fig. 5. Among three coping styles, the mediating role between Stress W1 and Anxiety W2 plays EOC W2 (statistical significance for standardized indirect effect is *p* < 0.001, 95% BCa *CI*_*B*_ = 0.253, 0.537). Higher stress during W1 can increase anxiety during W2 of the pandemic if people frequently use emotion-oriented coping. In yhe reciprocal direction, the mediating role between anxiety during W1 and stress during W2 seems to play TOC W2, but bootstrapping did not confirm this effect (standardized indirect effect *p* = 0.179, 95% BCa *CI*_*B*_ = − 0.031, 0.168). It seems that highly anxious individuals could slightly decrease stress levels if they implement task-oriented coping, but this effect is weak. Avoidance coping style (AOC W2) is negatively related to anxiety during W2, but is unrelated to stress W1, so its mediating role in the stress-anxiety interaction was not found in the study. Taking into account autoregressive path, the relationships between Stress W1 and Stress W2 is partially mediated by TOC W2 (negative association, standardized indirect effect *p* = 0.002, 95% BCa *CI*_*B*_ = − 0.191, 0.430) and EOC W2 (positive association, standardized indirect effect *p* = 0.001, 95% BCa *CI*_*B*_ = 0.210, 0.459). However, the mediating role of any coping styles was not found for the Anxiety W1 – Anxiety W2 relationship.

Fit indices for the Model 1 were unacceptable taking into account χ^2^/*df* = 12.082, and RMSEA = 0.227, but acceptable using CFI = 0.947 and SRMR = 0.096 [43]. Therefore, we removed AOC W2 in Model 2 and checked the goodness of fit again (Table 4). The fit of Model 2 improved significantly after removing AOC W2, with excellent indices, including χ^2^/*df* = 2.263, CFI = 0.998, and SRMR = 0.030, while acceptable RMSEA = 0.077.

**Table 3:** The standardizedized regression coefficients for variables in the model

Predictor		Outcome	*B*	*SE B*	β
Stress W1	→	Anxiety W1	0.469	0.028	0.756***
Stress W1	→	TOC W2	–0.571	0.138	–0.413***
Anxiety W1	→	AOC W2	0.169	0.197	0.087
Anxiety W1	→	EOC W2	0.226	0.233	0.093
Anxiety W1	→	TOC W2	0.514	0.223	0.231*
Stress W1	→	EOC W2	0.448	0.145	0.299**
Stress W1	→	AOC W2	0.188	0.123	0.156
AOC W2	→	Anxiety W2	–0.066	0.029	–0.116*
EOC W2	→	Anxiety W2	0.262	0.024	0.572***
TOC W2	→	Anxiety W2	–0.011	0.026	–0.022
Anxiety W1	→	Anxiety W2	0.284	0.085	0.257***
Stress W1	→	Anxiety W2	–0.002	0.055	–0.003
AOC W2	→	Stress W2	0.027	0.031	0.035
EOC W2	→	Stress W2	0.168	0.032	0.272***
TOC W2	→	Stress W2	–0.175	0.027	–0.261***
Anxiety W2	→	Stress W2	0.676	0.072	0.501***
Stress W1	→	Stress W2	0.066	0.058	0.071
Anxiety W1	→	Stress W2	0.040	0.092	0.027

*TOC* task-oriented coping,* EOC* emotion-oriented coping,* AOC* avoidance-oriented coping,* W1* wave 1 of the COVID-19 pandemic,* W2* wave 2 of the COVID-19 pandemic

*p < 0.05, **p < 0.01, ***p < 0.001

**Table 4:** Fit indices for Model 2

Model	χ^2^ (*df*)	*p*	SRMR	RMSEA	CFI
Baseline	2.263 (1)	0.132	0.025	0.077	0.998
Women	0.941 (1)	0.332	0.026	0.000	1.000
Men	1.455 (1)	0.228	0.026	0.061	0.999
Unconstrained	2.396 (2)	0.302	0.026	0.030	0.999
Constrained	20.189 (16)	0.212	0.069	0.035	0.993

* SRMR* standardized root mean square residual,* RMSEA* root mean square error of approximation,* CFI* comparative fit index

## Gender differences in the relationships between anxiety, stress, and coping

A multigroup SEM (MG SEM) was performed to examine hypothesis H4, whether a mediating effect of EOC and TOC on the stress-anxiety interaction is the same across genders (gender is considered as a moderator variable). Fit indices of the MG SEM Model 2 (without AOC W2) for the baseline, women, men, and multigroup comparison unconstrained and constrained, are presented in Table 4. No significant differences were found between unconstrained and constrained models, which means that the gender invariance assumption was confirmed. The same conclusion is derived from the analysis of path differences between women and men (Table 5). All gender differences in β are small and insignificant.

The indirect effect of perceived stress W1 on anxiety W2, via EOC W2 was significant for women (*p* < 0.01, 95% BCa *CI*_*B*_ = 0.107, 0.616), as well as for men (*p* < 0.001, 95% BCa *CI*_*B*_ = 0.251, 0.666). However, the indirect effect of anxiety W1 on perceived stress W2 via TOC W2 was significant for men (*p* < 0.05, 95% BCa *CI*_*B*_ = 0.023, 0.385), but insignificant for women (*p* = 0.900, 95% BCa *CI*_*B*_ = − 0.210, 0.231). Considering the autoregressive path, the association between Stress W1 and Stress W2 was mediated by TOC in both men (*p* = 0.001, 95% BCa *CI*_*B*_ = 0.142, 0.474) and women (*p* = 0.011, 95% BCa *CI*_*B*_ = 0.083, 0.496), and also the mediating role of EOC was confirmed in men (*p* = 0.001, 95% BCa *CI*_*B*_ = 0.177, 0.491) and in women (*p* = 0.008, 95% BCa *CI*_*B*_ = 0.091, 0.524).

**Table 5:** Standardized regression coefficients for variables in Model 2 by gender

Predictor		Outcome	Women β	Men β	*D* β	*D p*
Stress W1	→	Anxiety W1	0.756***	0.733***	0.023	1.000
Stress W1	→	TOC W2	–0.493**	–0.355**	–0.138	1.000
Anxiety W1	→	EOC W2	–0.087	0.223†	–0.311	1.000
Anxiety W1	→	TOC W2	0.289†	0.186	0.103	1.000
Stress W1	→	EOC W2	0.293†	0.289*	0.004	1.000
EOC W2	→	Anxiety W2	0.561***	0.528***	0.032	1.000
TOC W2	→	Anxiety W2	–0.05	–0.039	–0.011	1.000
Anxiety W1	→	Anxiety W2	0.255†	0.244*	0.011	1.000
Stress W1	→	Anxiety W2	–0.106	0.026	–0.132	1.000
EOC W2	→	Stress W2	0.252**	0.367***	–0.115	1.000
TOC W2	→	Stress W2	–0.206**	–0.304***	0.098	1.000
Anxiety W2	→	Stress W2	0.485***	0.476***	0.009	1.000
Stress W1	→	Stress W2	–0.027	0.107	–0.134	1.000
Anxiety W1	→	Stress W2	0.175	–0.101	0.276	1.000

*TOC* task-oriented coping,* EOC* emotion-oriented coping,* AOC* avoidance-oriented coping,* W1* wave 1 of the COVID-19 pandemic,* W2* wave 2 of the COVID-19 pandemic, *D* β = difference in betas, *D p* = p value for difference. †*p* < 0.10, *p < 0.05, **p < 0.01, ***p < 0.001

### Discussion

The first hypothesis, H1, that people constantly changed their coping strategies, adapting to unstable environments and their current mental state during the COVID-19 pandemic was partially confirmed. This study found significant differences in stress and two coping styles (TOC and AOC) between W1 and W2. Evidence indicates that TOC decreased during the second pandemic wave while stress increased. Also, the frequency of using AOC was reduced significantly at W2. In contrast, no significant changes were found in anxiety level and EOC between W1 and W2. It suggests that university students adaptively changed TOC and AOC coping strategies due to dynamic stress level changes. These results are consistent with the MIMSAC [2].

Gender differences were not fully confirmed in the study. Consistent with hypothesis H2 and previous research [2, 4, 13], women scored systematically higher than men in stress and anxiety. They also used more frequent AOC and EOC when compared to men. This study shows, however, no evidence for gender differences in using TOC. Furthermore, the interaction effect was not found currently. Previous research indicates that men use TOC more frequently than women [2, 4], which is inconsistent with the present results. TOC is usually seen as the most effective and adaptive coping style when the situation can be changed, controlled, or managed by an individual, so men and women have used it equally often during T1. However, during T2, the frequency of using TOC dropped significantly in both men and women. The COVID-19 pandemic statistics showed [39] that the number of new coronavirus cases and death in Poland significantly increased from W1 to W2, while the restriction level decreased. The Polish government’s response to the pandemic was inadequate to the situation, with exorbitant restrictions in the early phase of the pandemic while likely too low-level restrictions were administered during the second pandemic wave. Most likely, the TOC did not imply a more prolonged efficacy as the situation was unpredictable and uncontrolled due to the constant changes in the levels of restriction and the number of new cases of coronavirus infections and deaths.

The results indicate that higher stress levels are related to higher anxiety, which is in line with previous research [2, 4, 5]. Respondents who scored high in stress were more likely to use EOC and AOC while less frequently used TOC at W1 and W2. Also, anxiety correlated positively with EOC and AOC, while it seems not related to TOC in both pandemic waves. These associations are consistent to a great extent with previous studies [2, 4, 6–12]. Endler [2] argued that EOC is most effective in unpredictable situations, unlike TOC strategies, which seem not helpful. Therefore, we can conclude that this study’s association pattern is an adaptive stress response. It is important to note that frequent use of emotion-oriented coping strategies is more likely in “emotional people,” namely neurotics, who tend to react with high levels of negative emotions (e.g., frustration, anger, anxiety, stress) in stressful events. However, task-, emotion- and avoidance-oriented strategies are usually used simultaneously, interacting with each other to cope with stress and anxiety in the best way [48].

A prospective mediating effect of coping styles on the reciprocal relationship between stress and anxiety was confirmed in this study to some extent. Hypothesis H3 was supported partially since only EOC W2 has been recognized as a mediator in the perceived stress-anxiety association in both genders, while TOC W2 played mediating role in the anxiety-stress relationship, but it is true solely in men. However, no reciprocal direction was found for EOC W2 or TOC W2, neither AOC W2 was found as a mediator in the stress-anxiety interaction. The study suggests that anxiety could be reduced effectively by the frequent use of TOC in stressful situations during the COVID-19 pandemic. In contrast, people who frequently use EOC elevate anxiety during this highly stressful event. Furthermore, the autoregressive association between stress at W1 and W2 showed that people who frequently use EOC are progressively more stressed, while those often using TOC can be less stressed in a prospective time at W2 of the COVID-19 pandemic. Therefore, EOC seems to be an inadequate response to perceived stress and a harmful coping strategy, while TOC seems to be an adapting coping style, which plays a key role in decreasing stress.

Hypothesis H4, that there are gender differences in associations between variables was confirmed to some extent, but more research is needed to explain the inconsistency in the results. In general, the constrained MG SEM model did not differ significantly from the unconstrained, showing multigroup invariance. Consistent with this result, the mediating role of emotion-oriented coping style on the relationship between perceived stress W1 and anxiety W2 was significant for both genders. However, task-oriented coping can play a mediating role between anxiety W1 and perceived stress W2 only in men but not in women. Although the frequency of TOC use did not prevail among men, it seems that for males this coping style plays a key role in controlling the stress response and reducing its level. On the other hand, results of autoregressive path analysis demonstrate that EOC increases stress while TOC decreases it, and this effect is equal for both genders.

### Limitations of the study and future directions

There is some limitation that does not allow for the generalization of this study’s results. First of all, the self-report measures of stress, anxiety, and coping styles may be biased to some extent. Participants may choose a more socially acceptable response rather than being honest to avoid a negative image or may not be able to assess themselves accurately due to their poor introspective ability or the robust defense mechanisms they use. Further studies could use experimental methods to assess stress response and anxiety, such as physiological methods, like breathing assessment via capnometry, adrenal assessment, skin temperature, skin conductance, sleep tracking, resting heart rate, passive heart rate, heart rate variability (HRV), and brainwaves via electroencephalograph (EEG). Second, although all measures were performed during COVID-19, none included specific pandemic-related circumstances. Future studies may use more specific tools focused on the COVID-pandemic stressful event. Third, the findings were collected at one technical university in one country using an online survey, which may be related to the selection bias. Therefore, the results of the study cannot be generalized to the whole university student population. Future studies may consider the dissemination of online questionnaires using a university mailing list or paper-and-pencil methods of conducting research on many various types of universities across the country (e.g., humanistic, technical, art, music, higher vocational schools). Measures of income or socioeconomic status were not included in the study. Also, gender groups were not equal, with the predominance of men over women in the study. Future studies should include a more representative sample of university students and be more balanced regarding sociodemographic variables. Although we used a longitudinal design in this study, only two-time points were considered within the 6-month gap. Future research may assume more time points with shorter intervals. Also, it would be interesting to compare Bachelor’s and Master’s students in the future.

## Conclusion

The present study confirmed to some extent the MIMSAC. Research evidenced that coping strategies changed continually according to current stress and anxiety levels, playing an adaptive or maladaptive role at the present moment. The TOC was found as the most adaptive and efficient coping style in response to anxiety, which can significantly reduce stress, but solely in men. However, TOC do not play a mediating role in the relationship between stress and anxiety among women. On the other hand, stress was reduced successively in the second wave of the COVID-19 pandemic in those participants of both genders who used TOC. Furthermore, during the highly stressful situation related to the first wave of the COVID-19 pandemic, people systematically reduced TOC and AOC and increased EOC during the second pandemic wave. People, who implemented EOC as a response to perceived stress, increased their anxiety as well as stress levels. In the interaction between stress and anxiety, the vicious circle of negative emotion can lead to serious deterioration of mental health, increasing the risk of anxiety disorder, which can lead to depression. Therefore, EOC seems the strongest and most maladaptive coping style. Furthermore, the stress-anxiety interaction is not mediated by AOC, so avoidance seems ineffective during the COVID-19 pandemic.

Clinicians should suggest to their patients during the COVID-19 pandemic to use more frequently and in a wider range of task-oriented strategies (in particular among men) and to reduce the frequency of use of avoidance-oriented (as less effective) coping styles. Increased task-oriented coping can include positive thinking about solving everyday problems, creative thinking and using current resources in new ways, making plans and fulfilling them step by step, keeping a daily routine while lockdown and working from home and creating a place to work in the home instead of the office, involving others (colleagues from work, family members, friends), and assigning them tasks to do, managing time effectively. Especially, emotion-oriented coping strategies should be limited during the crisis of pandemics. Decrease in avoidance-oriented and emotion-oriented coping strategies can include eliminating or reducing negative emotions by reformulating the source of stress and problems by making them available for control and change, increasing optimism, positive emotions, and hope for a better future rather than avoiding COVID-19 problems by ignoring or suppressing them, or displacement with substitute activities. Current research showed that reappraisal as an emotion-regulation strategy effectively modifies how one thinks about a situation, reducing negative emotions and increasing positive emotions during the COVID-19 pandemic in participants from 87 countries and regions [49]. Previous research indicated that such strategies as disclosure and expressive writing might improve health and well-being [50, 51]. A meta-analysis also proved that benefit finding was related to less depression and more positive well-being [52]. Also, frequent use of positive emotion as a coping strategy was related to all positive aspects of well-being [53]. Therefore, reappraisal, benefit finding, disclosure, and expressive writing are recommended to reduce negative emotions, stress, and anxiety and improve wellbeing during the pandemic.

Moreover, knowledge of the current level of stress, anxiety, and preferred coping styles in patients is temporarily useful but needs to be reevaluated in subsequent pandemic waves due to constant changes in an uncontrolled or unpredictable environment. Since women scored higher than men in stress, anxiety, EOC, and AOC, the female gender should be considered a risk factor. Therefore, prevention and intervention programs for reducing negative emotions, stress, and anxiety should be primarily targeted at women, while men would benefit from learning and maintaining task-oriented strategies during the pandemic. Future research should focus on the mediating role of coping styles and their changes during the successive pandemic waves.

## Data Availability

All data have been made publicly available via Mendeley Data and can be accessed at Kuśnierz C, Rogowska A, Ochnik D. Stress, anxiety, and coping styles during the two waves of the COVID-19 pandemic. Mendeley Data, V1. 2021. https://data.mendeley.com/datasets/kmjs5hcwph/1.

## Abbreviations

ANOVA: Analysis of variance
AOC: Avoidance-oriented coping style
BCa: Bias-corrected percentile method of booptrapping
CI_B_: Bootstrap confidence interval
CFI: Comparative fit index
COVID-19: Coronavirus disease 2019
EOC: Emotion-oriented coping style
MIMSAC: Multidimensional interaction model of stress, anxiety and coping
MG SEM: Multigroup structural equation modelling
ML: Maximum likelihood estimation method
OUT: The Opole University of Technology
RMSEA: Root mean square error of approximation
SEM: Structural equation modelling
SRMR: Standardized root mean square residual
TOC: Task-oriented coping style
W1: The first wave of the pandemic
W2: The second wave of the pandemic
